# Attitudes towards persons with disabilities vs. personality traits of Polish students

**DOI:** 10.3389/fpsyt.2024.1477877

**Published:** 2025-01-27

**Authors:** Iwona Radlińska, Marta Kożybska, Arkadiusz Prajzner, Łukasz Krzywoszański, Beata Karakiewicz

**Affiliations:** ^1^ Subdepartment of Medical Law, Department of Social Medicine, Pomeranian Medical University in Szczecin, Szczecin, Poland; ^2^ Institute of Psychology, Faculty of Pedagogy and Psychology, University of the National Education Commission, Krakow, Poland; ^3^ Subdepartment of Social Medicine and Public Health, Department of Social Medicine, Pomeranian Medical University in Szczecin, Szczecin, Poland

**Keywords:** disability, attitudes, students, Poland, personality traits

## Abstract

**Objective:**

The study aimed to establish the relationship between attitudes towards persons with disabilities and personality traits among Polish students, and whether sociodemographic factors moderate this.

**Methods:**

A cross-sectional self-report online survey was conducted on 595 Polish students, aged 18–29, from different fields of study: 1) natural science and engineering technology; 2) social science and humanities; 3) medical and health sciences; 4) law, economics, and management. Polish adaptations of the scales were used in the study: Multidimensional Attitudes Scale Towards Persons With Disabilities (MAS) and the Ten Item Personality Inventory (TIPI).

**Results:**

A significant correlation was demonstrated between attitudes towards individuals with physical disabilities and a range of personality traits, including agreeableness, extroversion, conscientiousness, and openness, among Polish students. The strongest attitude predictors were openness to experience, agreeableness, and extraversion, with correlation coefficients ranging from -0.06 to -0.19, -0.14 to -0.22, and -0.09 to -0.15, respectively. As scores increased in these personality traits, attitudes towards people with disabilities became more positive. However, conscientiousness (-0.07 to -0.09) and emotional stability (-0.02 to 0.12) were poor predictors. The supplementary analyses indicate that contact with a person with a disability and socio-demographic factors, such as gender, age, place of residence, mode, and field of study, assessment of one’s health, and material conditions, did not moderate the relationships between personality dimensions and attitudes towards persons with disability.

**Conclusion:**

Polish students’ attitudes towards individuals with physical disabilities are associated with personality traits such as agreeableness, extraversion, conscientiousness, and openness. However, the strength of these relationships is relatively weak. This relationship is not moderated by contact with a person with a disability or sociodemographic factors. It seems that undertaking educational interventions to strengthen the indicated personality traits is particularly important. The results indicate the need for further research into factors that modulate attitudes towards persons with disabilities, including a theoretical deepening of the problem and cultural aspects.

## 1 Introduction

One of the groups particularly vulnerable to social isolation are persons with disabilities. According to the preamble of the United Nations Convention on the Rights of Persons with Disabilities from 6 December 2006 (CRPD Convention), ‘disability results from the interaction between persons with impairments and attitudinal and environmental barriers that hinders their full and effective participation in society on an equal basis with others’ (1). In this social conception, disability is not problematic in itself, but can become so in the interaction with certain social and environmental conditions. This perspective is implemented in recent World Health Organisation (WHO) policy documents. According to the WHO’s Disability Policy 2020, it emphasizes the need for countries and societies to actively remove these barriers in order to promote equality and inclusion of persons with disabilities (2). Undoubtedly, the change of the negative image has been and is being pursued by persons with disabilities themselves, who above all oppose being treated only as passive individuals in need of social services. They express their demands through the non-governmental organisations (NGOs) they create, which is called identity politics (3). For a very long time, it was thought that the most important barrier to access to civil society for people with disabilities was the removal of environmental (architectural) barriers. Still, over time, it became apparent that an equally important barrier was the mental one. The marginalisation of people with disabilities had its main source in an incorrect social perception, in which these people were treated as victims of fate (sad, unhappy people) and, over time, due to the development of social policy, as recipients of benefits (4). Attempts to remedy this situation have been made at various levels – changes to the names and content of diagnostic classifications, changes to the terminology and nomenclature of disabilities, and the introduction of provisions enabling inclusion in the fields of education, work, and culture. However, neither changes in terminology nor regulations have been sufficient without a change in the social mentality and therefore a change in the attitudes of all those surrounded by people with disabilities. It is thus emphasised that a positive image of people with disabilities should be continuously sought by raising awareness of disability and of the opportunities for their participation in society and by combating negative attitudes: stereotypes and prejudices (Article 8 CRDP: Awareness-raising) (1).

In social psychology, there are several theories on the structure of attitudes, i.e. how they are related in the human mind to thoughts, feelings, and behaviour. Among these is the Three-Component Model, which examines the dimensionality of attitudes – that is, how attitudes summarise either positive or negative attitudes towards an attitude object. The model suggests that people have a positive attitude towards an object when their beliefs, feelings, and behaviours express a favourable attitude towards the object (5). The Multidimensional Attitudes Scale Towards Persons With Disabilities (MAS) by Findler et al. (6), used to measure attitudes in our study, is based precisely on the Three-Component Model. In this view, attitude refers to feelings, beliefs, and reactions of an individual towards an event, phenomenon, objects, or person (7).

It can be concluded that the environment, culture, beliefs, emotions, feelings, perception, values, knowledge, information and experiences are consistent with an individual’s attitudes (7). In view of this, modulation of these factors can lead to a change in attitudes. Various theories explain the mechanisms of attitude formation and change, focusing on factors such as learning, self-observation, needs, group conformity, cognitive tension, and personal contact, as shown in **Table 1**, which contains theories of attitudes based on the available literature (7–10). Albert Bandura’s social learning theory is listed first in **Table 1** as a theory emphasising the key role of observation, modelling, and reinforcement (reward and punishment) in shaping attitudes and combining behavioural and cognitive elements. Undoubtedly, the influence of the life environment - primarily parents and the peer environment, is very strong in the formation of attitudes (8).

**Table 1:** Theories of attitudes based on available literature.

Theory	Modulation factors	Description
Social Learning Theory	Observation, modelling, reinforcement (rewards and punishments)	Attitudes are formed by observing behaviours and the consequences of actions demonstrated by social models, such as parents, peers, or media
Learning Theory	Participation in activities and observation of positive models	Assumes that attitudes can be modelled in a formalised learning process both through participation in activities and observing positive, exemplary behaviour
Self Perception Theory	Observation of one’s own behaviour	Suggests that individuals observe their behaviour and then change their attitudes so that they are consistent with their behaviour.
Functional Theory	Alignment of attitudes with personal needs or goals	Suggests that individuals adopt attitudes that match their needs.
Conformity Theory	Social influence and group norms	States that individuals act and think like the members of the group to which they belong
Cognitive Dissonance Theory	Discrepancy between beliefs self-image and behaviour	Describes dissonance as an unpleasant tension caused by conflicting beliefs or behaviours. To reduce this discomfort, individuals are motivated to change or rationalise their attitudes, beliefs, or actions.
Contact Theory	Direct interpersonal contact	Argues that personal interaction with individuals with disabilities fosters positive attitudes towards them.

Source: Own elaboration based on literature (7–10).

This brief overview of attitude theory suggests that modulation of attitudes can occur in a variety of ways, through changes in behaviour, needs, and environment, particularly through the provision of relevant knowledge and experience. Attitudinal theories can also explain the genesis of negative attitudes, such as stereotypes and prejudice. Both represent a superficial, simplified, and often irrational view of reality, with the difference that prejudice is always negative and can lead to hostile reactions (discrimination, aggression, violence) (11).

In particular, knowing a person with a disability in combination with extensive information about the disability (Contact Theory) is the realisation of several attitudinal theories, primarily Learning Theory, but also Conformity Theory (**Table 1**). Social inclusion of people with disabilities early, such as at the stage of school, can effectively change attitudes towards persons with disabilities, including the elimination of stereotypes and prejudices, and enable inclusion in society in adult life.

The study of personality distinguishes between different sets of constructs and measures, where there is agreement on the main dimensions of personality. Global traits such as neuroticism–stability, extraversion–introversion, psychoticism–constraint neuroticism–stability, extraversion–introversion, and psychoticism–constraint are identified in most personality trait analyses. Models based on trait concepts assume that traits vary along the dimension of breadth or generalisability and that traits are organised hierarchically, with global traits divided into a set of more specific traits, such as anxiety and dependence (12). Within this framework, attention has focused in particular on five main factors as a parsimonious taxonomy of personality traits (Goldberg, 1990), usually defined in five dimensions: extraversion or chirality (a), agreeableness (a), conscientiousness (c), emotional stability versus neuroticism (d), and intellect, culture or openness (e). These dimensions, called the Big-Five personality dimensions, are used in the tool used in our study. The Big-Five framework enjoys considerable support and has become the most widely used and extensively researched model of personality (Extraversion, Neuroticism, Conscientiousness, Agreeableness, and Openness – negatively) (9).

It should be added that, according to the behavioural genetic approach, there is evidence that genetic influences account for about 40–60% of the variance for virtually all personality traits. By contrast, most of the remaining variance is explained by environmental effects. On the one hand, it is emphasised that genetic influences are decisive here (12), while on the other hand, the relationship between genes and environment is still under investigation (13). This means that there is little chance of radically changing personality traits to, e.g., turn an extrovert into an introvert and vice versa, although environmental influences can modulate (strengthen or weaken) these traits. Personality traits can therefore be used in a positive sense as predispositions to perform certain professions or other social roles, while at the same time modulating them with an environmental factor to ensure the well-being and successful social functioning of the individual. It should be added that the impact of environmental influences on personality is widely discussed, which is undeniable, even if it does not involve intentional and targeted actions. Moreover, environmental influences can affect individual personality change in every period of life (14). Thus, environmental modulation is highly advisable and particularly relevant given the likely link between attitudes towards persons with disabilities and personality traits.

In terms of personality traits and attitudes towards persons with disabilities, there are few studies, including those concerning the area of intellectual disability (15, 16). Higher levels of openness and agreeableness were associated with more positive attitudes. A study by Page and Islam (16) confirmed the hypothesis that higher levels of the personality dimensions openness and agreeableness were significantly associated with positive attitudes towards persons with intellectual disabilities. However, the effect was relatively weak, and the strongest predictor of positive attitudes was higher contact quality (which meant having a previous positive contact experience) (16). Also, in the study by Himmelberger et al., openness to experience and agreeableness were significant predictors, although contact quality was the strongest. Further mediation analysis showed that contact quality mediated the relationship between openness and agreeableness, and attitudes (15).

Because of the scarcity of research on personality traits and attitudes towards people with disabilities, the subject of this study was the relationship between these traits and the attitudes of Polish students. We were also interested in and measured a set of variables identified as possible determinants of attitudes and personality traits. These variables included: gender, field of study, self-assessment of health and financial situation, and the fact of having contact with persons with disabilities.

The study aimed to assess the correlation between the personality traits of Polish students and their attitudes towards persons with disabilities. Additionally, the study aimed to identify any moderators of this relationship. Socio-demographic and education-related variables were also analysed to assess their possible interaction with personality traits in predicting attitudes towards persons with disabilities. The study’s aim and theoretical rationale led to the development of the following hypotheses:

1. There is a relationship between personality traits and attitudes towards persons with disabilities among Polish students.
2. The relationship between personality traits and attitudes towards persons with disabilities among Polish students is moderated by sociodemographic factors.

## 2 Materials and methods

### 2.1 Study procedure and participants

The research method was a cross-sectional diagnostic survey conducted between December 2021 and the end of April 2022, using the CAWI (Computer-Assisted Web Interview) method. The inclusion criteria for the survey were status as a Polish student, age between 18 and 29, and no disability. Students from all over Poland were able to complete the questionnaire via panelariadna.pl and via Microsoft Forms. Purposive sampling was used to obtain a similar number of students from different groups of fields of study: 1) technical (science, technology, natural sciences); 2) social (humanities and social sciences); 3) medical (medical and health sciences); and 4) administrative (law, economics, management and administration) (**Table 1**). Each student consented to participate in the study before completing the questionnaire and was informed about the purpose of the study and the possibility of withdrawing at any stage. The survey was self-reported.

### 2.2 Participants

Out of 669 respondents who completed the survey, only 595 responses were the subject of the analysis. Respondents who were over 29 years of age (*n* = 4), non-students (*n* = 7), individuals without Polish citizenship (*n* = 7), and those who reported having a disability (*n* = 56) were excluded from the analysis. We presented detailed sociodemographic characteristics of the study group in our previous article (17).

Respondents ranged in age from 18 to 29 years (*M* = 22.42; *SD* = 2.69). Females were the majority of the sample, accounting for over 71% of the participants. Over 75% of the sample were students residing in urban areas, with medicine being the most frequently indicated field of study. In addition, more than half of the participants reported having had contact with a person with a disability.

### 2.3 Measures

#### 2.3.1 Multidimensional attitudes scale towards persons with disabilities

The study used the Polish adaptation of the Multidimensional Attitudes Scale Towards Persons With Disabilities scale by Findler et al. (3, 6). The MAS-PL scale by Radlińska et al. (3) is used to assess attitudes towards persons with disabilities. The respondent is asked to imagine various situations involving a person in a wheelchair and to indicate the intensity of emotions (list of 16 emotions), thoughts (list of 10 thoughts), and potential behaviours (list of 8 behaviours) that a person without a disability involved in these situations might experience. The respondent indicates answers from 1 to 5, where 1 means ‘not at all’ and 5 ‘very much’. Positive items require reverse scoring. The higher the score, the greater the intensity of negative attitudes towards persons with disabilities. In addition to the overall score, the scale makes it possible to distinguish the area of emotions, beliefs, and behaviours. The reliability of the total score as well as of the three components in each case exceeds α = 0.80 (3).

#### 2.3.2 Ten item personality inventory

The tool by Sorkowska et al. (18) is an adaptation of a questionnaire developed by Gosling et al. (19), measuring the so-called ‘Big Five’ personality traits. The Big Five personality model, first proposed by John and Srivastava (20), includes five general personality factors: openness to experience, conscientiousness, extraversion, agreeableness, and neuroticism. In the Polish version, ‘neuroticism’ was replaced by the opposite term: ‘emotional stability’, in line with the approach of Golsing et al., who transformed the Big Five Inventory (BFI) into the Ten Item Personality Inventory (TIPI) with a 10-item measure of the Big-Five personality dimensions (19). The TIPI-PL inventory contains two pairs of statements relating to one of the traits under study. Respondents mark their answers on a Likert scale from 1 to 7, with 1 meaning ‘strongly disagree’ and 7 meaning ‘strongly agree’. One of the questions in each pair is reverse coded. The resulting score indicates the severity of each personality area. The reliability of the tool is moderate to good, showing the lowest Cronbach’s Alpha scores for openness to experience (0.44–0.47) and the highest for emotional stability (0.65–0.83).

#### 2.3.3 Custom questionnaire

Students were asked questions about socio-demographic data (age, gender, field of study, year and level of study, nationality), self-assessment of their financial situation (on a scale of 1–5; 1 – very bad, and 5 – very good), self-assessment of their health (on a scale of 1–5; 1 – very bad, and 5 – very good), whether they know a person with a disability personally and whether the participant has a disability themselves.

### 2.4 Statistical analysis

A significance level of 0.05 was chosen for the study. Raw personality scale scores were categorised as low, medium-low, medium-high and high, using quartiles. Our groups, identified by their TIPI scores, were compared across all MAS subscales using Multivariate Analysis of Variance (MANOVA), a statistical method that assesses differences across multiple dependent variables simultaneously while accounting for potential intercorrelations among them. MANOVA ensures that the probability of Type I errors is controlled when multiple outcome variables are considered together. The multivariate tests were evaluated using Wilks’ Lambda, a coefficient that ranges from 0 to 1. Wilks’ Lambda quantifies the proportion of total variance in the dependent variables not explained by the independent variable(s). Values close to 0 indicate a strong multivariate effect, while values closer to 1 suggest weaker multivariate effects. In practice, a Wilks’ Lambda value below 0.90 is often considered to suggest at least a small effect, but precise thresholds depend on context and sample size. When significant multivariate effects were observed, follow-up analyses were conducted to better understand the specific sources of variation. Univariate Analysis of Variance (ANOVA) was performed on each MAS subscale individually to identify which dependent variables contributed to the significant multivariate effect. This analytic strategy aligns with best practices in multivariate statistical analyses (21, 22). For cases where ANOVA yielded significant results, *post-hoc* pairwise comparisons were carried out using Tukey’s Honestly Significant Difference (HSD) test (23), ensuring control of the family-wise error rate. Effect sizes were assessed using partial eta-squared, which quantifies the proportion of variance in each dependent variable explained by group membership. Effect sizes were interpreted according to established thresholds, with values of 0.01, 0.06, and 0.14 classified as small, moderate, and large effects, respectively (24). Sociodemographic factors were analysed using general linear models, while linear multivariate regression models were used to assess prediction after early testing and rejection of curvilinear relationships. The study results were processed using TIBCO Software’s Statistica 13.3, and Jamovi 2.4.14.

## 3 Results

The results of MANOVA multivariate tests are presented in **Table 2**. They indicate that the four groups, distinguished by the levels of Extraversion, Agreeableness, Conscientiousness, Emotional stability, and Openness to experience, differed in their scores on MAS subscales.

**Table 2:** Results of multivariate tests of differences in subscales of the Multidimensional Attitudes Towards Persons with Disabilities (MAS) between four groups, constructed based on categorised scores from the Ten Item Personality Inventory (TIPI).

Statistic	TIPI results highlight groups				
	Extraversion	Agreeableness	Conscientiousness	Emotional stability	Openness to experience
*Wilk’s Λ*	0.88	0.89	0.94	0.96	0.90
*Df_Hipotesis_*	9	9	9	9	9
*Df_Error_*	1433.62	1433.62	1433.62	1433.62	1433.62
*F*	8.23	7.60	4.37	2.69	6.79
*p*	<0.001	<0.001	<0.001	0.004	<0.001
*η^2p^*	0.04	0.04	0.02	0.01	0.03

*Df*, degrees of freedom; *F*, test statistic for MANOVA; *p*, significance; *η^2p^*, partial eta squared.

The results of univariate tests for differences in the MAS subscales among the Extraversion groups performed using one-way ANOVA and the results of the one-way ANOVA for MAS total scores are shown in **Table 3**.

**Table 3:** Comparisons of the Multidimensional Attitudes Towards Persons with Disabilities (MAS) scores between the four groups, differing in their scores on the Extraversion scale of the Ten Item Personality Inventory (TIPI).

MAS scores	Extraversion								*F_(3, 591)_*	*p*	*η^2p^*	*post hoc*
	Low (1) *N* = 142		Mid-low (2) *N* = 119		Mid-high (3) *N* = 198		High (4) *N* = 136					
	*M*	*SD*	*M*	*SD*	*M*	*SD*	*M*	*SD*				
Emotions	2.88	0.58	2.82	0.63	2.65	0.62	2.55	0.66	12.98	<0.001	0.06	1, 2>3, 4
Beliefs	2.57	0.67	2.39	0.63	2.32	0.71	2.14	0.69	10.18	<0.001	0.05	1, 2>3, 4
Behaviours	2.75	0.68	2.56	0.66	2.42	0.68	2.24	0.72	10.49	<0.001	0.05	1, 2, 3>4
Total	2.76	0.46	2.63	0.46	2.50	0.48	2.36	0.53	19.61	<0.001	0.09	1, 2>3>4

*Post-hoc* analysis was performed using Tukey’s honestly significant difference test.

MAS, Multidimensional Attitudes Scale Towards Persons With Disabilities; *N*, number of observations; *M*, mean of scores; *SD*, standard deviation; *F*, test statistic for ANOVA; *p*, significance; *η^2p^*, partial eta squared.

The analyses revealed that extraversion significantly differentiated attitudes towards persons with disabilities, in terms of both the total score and specific domains. The effects were weak and moderate for the subdomains and the total score. The results of the *post-hoc* pairwise comparisons indicate that groups with low and mid-low levels of extraversion exhibited significantly higher and more negative attitudes towards persons with disabilities than the group with mid-high and high extraversion.

**Table 4** summarises the comparisons of attitudes towards persons with disabilities between groups that differ in agreeableness.

**Table 4:** Comparisons of the Multidimensional Attitudes Towards Persons with Disabilities (MAS) scores between the four groups, differing in their scores on the Agreeableness scale of the Ten Item Personality Inventory (TIPI).

MAS scores	Agreeableness								*F_(3, 591)_*	*p*	*η^2p^*	*post hoc*
	Low (1) *N* = 181		Mid-low (2) *N* = 149		Mid-high (3) *N* = 96		High (4) *N* = 169					
	*M*	*SD*	*M*	*SD*	*M*	*SD*	*M*	*SD*				
Emotions	2.88	0.58	2.82	0.63	2.65	0.62	2.55	0.66	9.70	<0.001	0.05	1>3, 4; 2>4
Beliefs	2.57	0.67	2.39	0.63	2.32	0.71	2.14	0.69	11.71	<0.001	0.06	1>3, 4; 2>4
Behaviours	2.75	0.68	2.56	0.66	2.42	0.68	2.24	0.72	16.70	<0.001	0.08	1>3, 4; 2>4
Total	2.76	0.46	2.63	0.46	2.50	0.48	2.36	0.53	21.65	<0.001	0.10	1, 2>3, 4

*Post-hoc* analysis was performed using Tukey’s honestly significant difference test.

MAS, Multidimensional Attitudes Scale Towards Persons With Disabilities; *N*, number of observations; *M*, mean of scores; *SD*, standard deviation; *F*, test statistic for ANOVA; *p*, significance; *η^2p^*, partial eta squared.

Similar to extroversion, agreeableness was found to significantly differentiate all attitude domains. However, the effects were also weak and moderate in the domains of emotions, beliefs, and behaviour. There was a moderate effect on the total score. The *post-hoc* evaluation revealed that the low agreeableness groups held significantly more negative attitudes towards persons with disabilities than the mid-high and high agreeableness groups. Furthermore, differences were observed between the mid-low and high levels of agreeableness.

Another personality trait analysed is conscientiousness, which is summarised in **Table 5**.

**Table 5:** Comparisons of the Multidimensional Attitudes Towards Persons with Disabilities (MAS) scores between the four groups, differing in their scores on the Conscientiousness scale of the Ten Item Personality Inventory (TIPI).

MAS scores	Conscientiousness								*F_(3, 591)_*	*p*	*η^2p^*	*post hoc*
	Low (1) *N* = 201		Mid-low (2) *N* = 72		Mid-high (3) *N* = 168		High (4) *N* = 154					
	*M*	*SD*	*M*	*SD*	*M*	*SD*	*M*	*SD*				
Emotions	2.85	0.59	2.81	0.60	2.69	0.63	2.58	0.68	6.15	<0.001	0.03	1, 2>4
Beliefs	2.48	0.71	2.45	0.67	2.36	0.66	2.17	0.68	6.76	<0.001	0.03	1, 2>3>4
Behaviours	2.68	0.68	2.56	0.71	2.45	0.70	2.31	0.73	8.71	<0.001	0.04	1>3, 4
Total	2.70	0.45	2.64	0.46	2.54	0.52	2.39	0.52	12.35	<0.001	0.06	1, 2>3>4

*Post-hoc* analysis was performed using Tukey’s honestly significant difference test.

MAS, Multidimensional Attitudes Scale Towards Persons With Disabilities; *N*, number of observations; *M*, mean of scores; *SD*, standard deviation; *F*, test statistic for ANOVA; *p*, significance; *η^2p^*, partial eta squared.

The personality trait of conscientiousness was found to significantly differentiate attitudes towards persons with disabilities in all specific domains, although the effects remained weak. The effect on the total score was moderate. Pairwise comparisons revealed that groups with low and mid-low conscientiousness had significantly higher negative attitude scores towards persons with disabilities than the group with mid-high and high conscientiousness. Furthermore, a significant difference was observed between the mid-high and high conscientiousness groups for the attitude total score.

**Table 6** summarises the differences in attitudes towards persons with disabilities between the groups distinguished by level of emotional stability.

**Table 6:** Comparisons of the Multidimensional Attitudes Towards Persons with Disabilities (MAS) scores between the four groups, differing in their scores on the Emotional stability scale of the Ten Item Personality Inventory (TIPI).

MAS scores	Emotional stability								*F_(3, 591)_*	*p*	*η^2p^*	*post hoc*
	Low (1) *N* = 176		Mid-low (2) *N* = 84		Mid-high (3) *N* = 201		High (4) *N* = 134					
	*M*	*SD*	*M*	*SD*	*M*	*SD*	*M*	*SD*				
Emotions	2.76	0.64	2.77	0.64	2.77	0.60	2.60	0.68	2.38	0.069	0.01	–
Beliefs	2.30	0.66	2.36	0.60	2.47	0.69	2.29	0.77	2.84	0.037	0.01	–
Behaviours	2.41	0.71	2.50	0.74	2.64	0.72	2.44	0.68	3.80	0.010	0.02	1<3
Total	2.54	0.50	2.59	0.48	2.65	0.49	2.47	0.53	3.78	0.011	0.02	3>4

*Post-hoc* analysis was performed using Tukey’s honestly significant difference test.

MAS, Multidimensional Attitudes Scale Towards Persons With Disabilities; *N*, number of observations; *M*, mean of scores; *SD*, standard deviation; *F*, test statistic for ANOVA; *p*, significance; *η^2p^*, partial eta squared.

Significant differences in the main attitude score, as well as in the belief and behaviour domains, were found between groups with different levels of emotional stability, although the effects were weak. High levels of emotional stability were associated with lower scores of negative attitudes towards persons with disabilities. In interpreting the results, a curvilinear trend is observed, indicating that attitudes towards persons with disabilities are more positive at low and high levels of stability, while the highest negative attitude scores are obtained at the mid levels.

The openness to experience was the last to be evaluated. **Table 7** provides a summary of this difference analysis.

**Table 7:** Comparisons of the Multidimensional Attitudes Towards Persons with Disabilities (MAS) scores between the four groups, differing in their scores on the Openness to experience scale of the Ten Item Personality Inventory (TIPI).

MAS scores	Openness to experience								*F_(3, 591)_*	*p*	*η^2p^*	*post hoc*
	Low (1) *N* = 103		Mid-low (2) *N* = 171		Mid-high (3) *N* = 213		High (4) *N* = 108					
	*M*	*SD*	*M*	*SD*	*M*	*SD*	*M*	*SD*				
Emotions	2.93	0.58	2.76	0.59	2.76	0.62	2.43	0.71	12.33	<0.001	0.06	1, 2,3>4
Beliefs	2.55	0.65	2.48	0.73	2.26	0.62	2.20	0.75	7.56	<0.001	0.04	1, 2>3,4
Behaviours	2.68	0.70	2.63	0.64	2.47	0.71	2.22	0.77	10.46	<0.001	0.05	1, 2,3>4
Total	2.76	0.45	2.65	0.46	2.55	0.49	2.31	0.55	16.79	<0.001	0.08	1>3>4; 2>4

*Post-hoc* analysis was performed using Tukey’s honestly significant difference test.

MAS, Multidimensional Attitudes Scale Towards Persons With Disabilities; *N*, number of observations; *M*, mean of scores; *SD*, standard deviation; *F*, test statistic for ANOVA; *p*, significance; *η^2p^*, partial eta squared.

In all domains of attitudes towards persons with disabilities, a significant differentiation was observed between various levels of openness to experience. The effects were described as weak and moderate. The *post-hoc* analysis revealed that the low and mid-low openness to experience groups exhibited higher scores on negative attitudes towards persons with disabilities than the mid-high and high openness to experience groups.

The data were also analysed using multivariate regression models, with the personality traits as the predictors and attitudes towards persons with disabilities as the dependent variables. The models that predicted the overall attitude score, emotion, belief, and behaviour domains were well-fitted to the data and explained 18%, 9%, 6%, and 12%, respectively. Personality traits accounted for most of the variation in the specific domains of attitudes towards persons with disabilities. Agreeableness emerged as a personality trait that significantly predicted each domain of attitudes, with a negative and weak relationship (-0.14 to -0.22). The study found that extraversion was significantly related to emotions, beliefs, and the main outcome, with negative and weak correlations (-0.11 to -0.15). Conscientiousness was a significant predictor in the model for predicting beliefs, behaviours, and the total attitude score. Conscientiousness also showed weak and negative correlations with attitudes (-0.08 to -0.09). Openness to experience showed significant negative correlations with emotions, behaviours, and overall outcome (-0.14 to -0.19). Emotional stability was a significant predictor only in the model predicting behaviour (-0.02 to 0.12). The results of the regression models are shown in **Table 8**.

**Table 8:** Results from four multivariate linear regression analyses to predict the Multidimensional Attitudes Towards Persons with Disabilities (MAS) scores from scores on the Ten Item Personality Inventory (TIPI) scales.

MAS scores	*F_(5, 589)_*	*R_s_^2^*	*p*	Predictor	*β*	95% *CI*		*p*
						*UL*	*LL*	
Emotions	13.51	0.09	<0.001	Extraversion	-0.11	-0.20	-0.02	0.019
				Agreeableness	-0.14	-0.22	-0.06	<0.001
				Conscientiousness	-0.07	-0.15	0.01	0.092
				Emotional stability	-0.02	-0.10	0.06	0.660
				Openness to experience	-0.17	-0.26	-0.09	<0.001
Beliefs	9.09	0.06	<0.001	Extraversion	-0.11	-0.20	-0.02	0.014
				Agreeableness	-0.16	-0.25	-0.08	<0.001
				Conscientiousness	-0.08	-0.16	0.00	0.048
				Emotional stability	0.05	-0.03	0.14	0.202
				Openness to experience	-0.06	-0.14	0.02	0.134
Behaviours	16.95	0.12	<0.001	Extraversion	-0.09	-0.19	0.00	0.056
				Agreeableness	-0.21	-0.29	-0.12	<0.001
				Conscientiousness	-0.09	-0.18	-0.01	0.030
				Emotional stability	0.12	0.04	0.21	0.004
				Openness to experience	-0.14	-0.22	-0.05	0.001
Total	27.10	0.18	<0.001	Extraversion	-0.15	-0.24	-0.06	0.001
				Agreeableness	-0.22	-0.30	-0.14	<0.001
				Conscientiousness	-0.08	-0.16	0.00	0.039
				Emotional stability	0.06	-0.02	0.14	0.161
				Openness to experience	-0.19	-0.27	-0.11	<0.001

Analysis was conducted for a set of 595 observations.

MAS, Multidimensional Attitudes Scale Towards Persons With Disabilities; *F*, ANOVA model fit test; *R_s_^2^*, coefficient of determination; *p*, significance; *β*, standardised coefficient; *CI*, confidence interval; *LL*, lower limit; *UP*, upper limit.

Upon assessing the effect sizes of the difference analyses and the strengths of the relationships in the correlation models, it is evident that openness to experience, agreeableness, and extraversion were the strongest predictors of negative attitudes towards individuals with disabilities. As scores on openness, agreeableness, and extraversion increased, attitudes towards individuals with disabilities were perceived to be more positive. Conscientiousness and emotional stability were among the weakest predictors of negative attitudes towards individuals with disabilities.

The appendix analyses, available in the Supplementary Material, indicate that socio-demographic factors, such as gender, age, place of residence, mode, and field of study, as well as the assessment of one’s health and material conditions, did not moderate the relationships between personality dimensions and attitudes towards persons with disabilities. Familiarity with persons with disabilities also did not moderate the relationship between personality dimensions and attitudes towards them. The relationship between students’ personality traits and attitudes towards persons with disabilities was not significantly moderated by sociodemographic factors.

## 4 Discussion

The Preamble of the CRPD Convention emphasises that the concept of disability is an evolving one, and thus it falls upon societies to eradicate any form of discrimination against persons with disabilities that violates their inherent dignity. Furthermore, it is acknowledged that discrimination against any individual on the basis of disability is a violation of their inherent dignity and worth as a human being (1). Consequently, the most crucial aspect in ensuring the inclusion of people with disabilities is the necessity to address and rectify the prevailing attitudes towards them, which can impede inclusion and even result in discriminatory practices, namely unequal and unfair treatment.

When defining attitudes, social psychologists focus on the tendency to like or dislike the object of the attitude, and thus attitudes can be favourable (positive attitudes) or unfavourable (negative attitudes). The attitude object can be any object in the environment, including groups of people, controversial issues and specific objects. One of the key attributes of attitudes is that they are subjective – that is, they reflect how a person sees an object and not necessarily how the object actually exists. Among the most important attributes of attitudes, we can mention the internal element related to a person’s identity and personality and the external element, which is the social experiences of that person (25).

Attitudes are a powerful determinant of human behaviour, so researchers have devoted much research to how people acquire them and under what circumstances they change (7). Research has shown that there are several ways in which attitudes are acquired and shaped. One of the first factors shaping attitudes is parents, followed by peers and the media. There is then mainly a mechanism of learning by observation and persuasion (urging a change of attitude, e.g. apologising for the harm done), and instrumental conditioning, i.e. being rewarded or punished for behaviours and attitudes. Attitudes are influenced by the moral evaluation a person makes (associating behaviours and attitudes with ‘good’ or ‘bad’ – classical conditioning) and by cognitive evaluation – weighing logical arguments to determine one’s attitudes. It should be added that attitudes can be very complex and vary according to personality, temperament (extrovert/introvert), knowledge and upbringing (rational and irrational attitudes), and self-awareness (conscious and unconscious attitudes – this heightened duality can lead to neurosis) (7).

In previous research on factors associated with attitudes towards persons with disabilities, many researchers have shown that work and non-professional experience with persons with disabilities (15–17, 26–31), female gender (11, 15, 16, 30, 32–38) (although not in all studies (11, 17, 39–41)), and among students, studying a healthcare (health-related) course (17, 26, 28, 36), are associated with favourable attitudes. Some studies point to the influence of age (16, 30, 42), race or ethnicity (26, 32), disability knowledge (31), and learning in an inclusive classroom (43). Attitudinal studies comparing the attitudes of first-year students, final-year students and medical professionals do not provide a clear answer to whether the role of student or professional is related to attitudes towards persons with disabilities, or whether completion of disability-related courses influences these attitudes (26, 32, 34, 37, 38, 44–46).

Most studies suggest that more contact with persons with disabilities generates more positive attitudes towards them (15, 16, 26, 27, 29).

Increased frequency of contact reduces prejudice by broadening perspectives, increasing empathy, and reducing intergroup threat and anxiety (47). Satchidanand et al. in a systematic review of attitudes of healthcare students and professionals toward patients with physical disability indicated that one of the factors associated with more positive attitudes toward persons with disabilities was the frequency of contact (26). It should be emphasized that some of the studies included in this review concerned only close contact (27, 48). However, Eberhardt et al. found that students who experienced a greater frequency of professional contact with persons with disabilities also seemed to experience more personal contact with these persons (49). Interestingly, research on attitudes towards individuals with intellectual disabilities indicates that the frequency of contact is a predictor of more positive attitudes only when combined with the quality of this contact (15, 50, 51). At the same time, the quality of the contact is an independent predictor of positive attitudes (15, 16, 50, 51). Also, educational interventions have been proven to have positive results in improving attitudes (27, 43). This regularity is called Contact Theory (9). The results of the 2020 systematic review indicate that educational interventions combining contact and information are associated with more positive attitudes among students (9). Meanwhile, in a study of students’ attitudes, which included an attitude survey before a contact intervention and one year after the intervention, Cecchetti et al. observed no significant association between levels of social contact and measures of attitudes and empathy (52). This study highlighted the difficulty of maintaining such attitude improvements, the benefits of which were sustained over the long term (52). Other researchers emphasise that the quality of contact as a predictor of attitudes is important, so careful preparation of educational interventions is recommended (15, 16, 51).

Positive attitudes in inclusive education as a first stage in the social life of people with different types of disabilities are particularly important. The results of Albuquerque et al. suggest that disability should be presented in a positive way, which increases the positive attitudes of parents of typically developing children (TDC) towards school inclusion education, and thus increases the success of this education (53). Also, the results of Krischler et al. (54) show that participants expressed generally positive attitudes towards inclusive education, but the attitudes of participants towards the inclusion of students with learning disabilities or challenging behaviour in mainstream classes were negative. Unfortunately, negative attitudes towards persons with learning disabilities are firmly entrenched (55). Recent research describes effective educational interventions that positively influence attitudes towards individuals with disabilities, e.g. the use of mixed methods (a mixed method training programme in improving attitudes) (56), role play (57), simulation-based, holistic health-care education (58), participation in integrated sport (59), and disability awareness training (60). Among other factors associated with more negative attitudes towards persons with disabilities, problematic use of the Internet (17), experiencing peer-rejected individuals in childhood with low support from parents (61), and lower levels of individual and classroom moral identity (43) were identified.

To date, only two studies have examined the relationship between attitudes towards individuals with disabilities and personality traits (15, 16). The inaugural study on this subject was published in 2015 by Page and Islam (16). In contrast to our study, the authors examined attitudes towards individuals with intellectual disabilities. The authors employed the Mental Retardation Attitude Inventory and the Big Five Inventory. The study group comprised 466 adults aged between 18 and over 70 years, with a preponderance of women, who were recruited by distributing an online survey on the Internet. The research was conducted in Australia. With regard to personality traits, the findings indicated that agreeableness and openness were predictive of more positive attitudes, albeit with a relatively weak effect.

The study by Page & Islam (16) was replicated by Himmelberger et al. in 2023 (15). The study was conducted on a sample of 221 undergraduate students in the United States, with a female predominance, using the Mental Retardation Attitude Inventory-Revised and the Big Five Inventory. The hypothesis that agreeableness and openness are predictors of attitudes towards persons with disabilities was confirmed.

In our study, the first hypothesis tested was that there is a relationship between personality traits and attitudes towards persons with physical disabilities among Polish students. This hypothesis was confirmed. It was shown that there is a relationship between attitudes towards persons with physical disabilities and such personality traits as agreeableness, extraversion, conscientiousness, openness to experience, and emotional stability among Polish students. Openness to experience, agreeableness, and extraversion were found to be the strongest predictors of attitudes. As scores increased in these personality traits, attitudes towards persons with disabilities were more positive. Conscientiousness and emotional stability proved to be weak predictors.

The use of the MAS scale, which distinguishes subscales of emotions, behaviours, and beliefs, enabled us to gain a broader understanding of the associations of personality traits with attitudes towards persons with physical disabilities. Thus, extraversion, agreeableness, openness to experience, and conscientiousness were associated with more positive attitudes towards individuals with disabilities in all areas of the MAS scale. The effects were weak and moderate. Emotional stability was associated with lower main attitude scores and lower scores in the areas of belief and behaviour (implying more positive attitudes). However, the effect was weak.

Thus, our own research showed a relationship of attitudes towards persons with disabilities not only with agreeableness and openness, as in previous studies (15, 16), but also with extraversion and conscientiousness. The differences may be due to the fact that different tools were used to measure attitudes and the studies concerned different types of disability.

The second hypothesis posed in the study assumed that the relationship between personality traits and attitudes towards persons with disabilities among Polish students is moderated by sociodemographic factors. This hypothesis was not confirmed. Variables such as gender, age, place of residence, mode, and field of study, as well as a subjective assessment of one’s own health and material conditions, did not moderate the relationships between personality dimensions and attitudes towards persons with disabilities (analyses are included in the Supplementary Materials). Interestingly, familiarity with disabled individuals also did not moderate this relationship. Different results were obtained by Himmelberger et al. In their study, contact quality mediated the relationships between openness, and agreeableness and attitudes (15). The differences may be due to the different methods used to measure the respondents’ contact with persons with disabilities.

### 4.1 Limitations

The use of self-report methods may also have been a limitation of the study. Questionnaire-based surveys always allow the respondents to tend towards social approval (known in psychology as social desirability). Social desirability (SD) may not only affect the answers to questions about attitudes towards persons with disabilities but also impact the results obtained in personality questionnaires, such as conscientiousness and extraversion scales since high levels of these traits are usually considered socially expected. SD reflects a respondent’s inclination to provide answers that align with prevailing social expectations and present themselves in a positive light. Such bias can significantly impact various psychological variables when assessed through self-report instruments (62). Given that self-report measures were central to this study’s methodology, it is crucial to further investigate the effects of SD in future research to better isolate genuine psychological constructs from response biases.

The lack of a moderating effect of familiarity with persons with disabilities on the relationship between personality traits and attitudes towards them may be due to potential limitations in the questionnaire used to assess these variables. Therefore, it is essential to verify the absence of the expected moderating effects using different tools to assess attitudes towards persons with disabilities. We also did not measure the quality of contact with persons with disabilities. We only had a question about whether you personally knew a person with a disability. Himmelberger et al. (15) and Page & Islam (16), in contrast, measured the quality and quantity of this contact with a Likert scale.

## 5 Conclusion

Polish students*’* attitudes towards persons with physical disabilities were associated with their personality traits. However, the strength of these relationships is relatively weak. This relationship was shown to exist with such personality traits as agreeableness, extraversion, conscientiousness, and openness. Openness to experience, agreeableness, and extraversion were found to be the strongest predictors of attitudes. As scores increased in these personality traits, attitudes towards persons with disabilities were more positive. Conscientiousness and emotional stability appeared to be poor predictors. Educational interventions, in the form of short and long training courses and even entire curricula at all levels of education, to strengthen personality traits associated with positive attitudes (openness to experience, agreeableness, and extraversion) seem to be particularly relevant. Personality formation also means, of course, encouraging children and young people to participate in various extracurricular activities (sports, arts), which can have a positive impact on their development – strengthening desirable qualities and/or weakening undesirable ones.

The relationship between personality traits and attitudes towards persons with disabilities among Polish students is not moderated by sociodemographic factors, such as gender, age, place of residence, mode, and field of study, as well as an assessment of one’s health and material conditions. Contact with persons with a disability did not moderate this relationship. Given the different results of the works of other authors, it is necessary to continue research in this area, especially to examine the impact of social desirability on respondents’ answers.

At the same time, the results we obtained, especially regarding unconfirmed hypotheses, indicate the need for further research on factors modulating attitudes towards persons with disabilities, including a theoretical deepening of the problem and cultural aspects. There is no doubt that as a society we are struggling with the exclusion of individuals with disabilities from many areas of life.

## Data Availability

The raw data supporting the conclusions of this article will be made available by the authors, without undue reservation.

## References

[1] United Nations. Convention on the Rights of Persons with Disabilities [A/RES/61/106] (2006). Available online at: https://www.un.org/esa/socdev/enable/rights/convtexte.htm (Accessed December 16, 2024).

[2] World Health Organization. WHO Policy on Disability. 1st ed. Geneva. (2021). Available online at: https://www.who.int/publications/i/item/9789240020627 (Accessed December 16, 2024).

[3] Radlińska I Starkowska A Kożybska M Flaga-Gieruszyńska K Karakiewicz B. The multidimensional attitudes scale towards persons with disabilities (Mas) – a polish adaptation (mas-pl). Ann Agric Environ Med. (2020) 27:613–20. doi: 10.26444/aaem/114531 33356069

[4] Kolwitz M Radlińska I. Development of a modern paradigm of disability. Pomeranian J Life Sci. (2015) 61:270–7. doi: 10.21164/pomjlifesci.99 27344868

[5] Olson JM Maio GR. Attitudes in social behavior, chapter 13. In: Handbook of Psychology, Volume 5, Personality And Social Psychology. John Wiley & Sons, Inc, Hoboken, New Jersey, USA (2003). p. 299–318. Available at: https://analytics.ng/wp-content/uploads/2020/04/Handbook-of-Psychology-Personality-and-Social-Psychology-Analytics.NG_.pdfpage=321 (Accessed December 16, 2024).

[6] Findler L Vilchinsky N Werner S. The multidimensional attitudes scale toward persons with disabilities (MAS). Rehabil Couns Bull. (2007) 50:166–76. doi: 10.1037/t48910-000

[7] Olufemi TD. Theories of attitudes, chapter 3. In: Psychology of Attitudes. New York (US): Nova Science Publishers, Inc (2012). p. 62–78.

[8] Bandura A. Social-learning theory of identificatory processes. In: Goslin A, editor. Handbook of socialization theory and research D. Rand McNally & Company, Chicago, IL (1969). p. 213–62.

[9] Rademaker F de Boer A Kupers E Minnaert A. Applying the contact theory in inclusive education: A systematic review on the impact of contact and information on the social participation of students with disabilities. (2020). doi: 10.3389/feduc.2020.602414

[10] O’Connor CMA. An Analysis of Leon Festinger’s A Theory of Cognitive Dissonance. London: Macat Library (2017). p. 97. doi: 10.4324/9781912282432

[11] Radlińska I Kożybska M Karakiewicz B. Attitudes of polish medical and health sciences students towards persons with physical disabilities using the mas-pl scale. Int J Environ Res Public Health. (2021) 18:7787. doi: 10.3390/ijerph18157787 34360078 PMC8345448

[12] Livesley WJ Jang KL Vernon PA. Genetic basis of personality structure, chapter 3. In: Handbook of Psychology, Volume 5, Personality And Social Psychology. John Wiley & Sons, Inc, Hoboken, New Jersey (2003). p. 59–79. Available at: https://analytics.ng/wp-content/uploads/2020/04/Handbook-of-Psychology-Personality-and-Social-Psychology-Analytics.NG_.pdfpage=321 (Accessed December 16, 2024).

[13] Balestri M Calati R Serretti A De Ronchi D. Genetic modulation of personality traits: a systematic review of the literature. Int Clin Psychopharmacol. (2014) 29:1. doi: 10.1097/YIC.0b013e328364590b 24100617

[14] Kandler C Papendick M. 29 - Behavior genetics and personality development: A methodological and meta-analytic review. In: Specht J, editor. Personality Development Across the Lifespan. Cambridge, Massachusetts (US): Academic Press (2017). p. 473–95. doi: 10.1016/B978-0-12-804674-6.00029-6

[15] Himmelberger ZM Faught GG Tungate AS Conners FA Merrill EC. Personality traits predict attitudes toward individuals with intellectual disability. Int J Dev Disabil. (2023) 69:906–14. doi: 10.1080/20473869.2022.2044594 PMC10599174 37885845

[16] Page SL Islam MR. The role of personality variables in predicting attitudes toward people with intellectual disability: An Australian perspective. J Intellect Disabil Res. (2015) 59:741–5. doi: 10.1111/jir.12180 25559160

[17] Kożybska M Radlińska I Prajzner A Krzywoszański Ł Karakiewicz B. Problematic Internet use and attitudes towards persons with disabilities – cross-sectional research among Polish students. BMC Med Educ. (2023) 23:915. doi: 10.1186/s12909-023-04816-x 38049791 PMC10696821

[18] Sorokowska A Słowińska A Zbieg A Sorokowski P. Polska adaptacja testu Ten Item Personality Inventory (TIPI) –TIPI-PL – wersja standardowa i internetowa (Polish adaptation of the TIPI Test- standard and web version). Wrocław: WrocLab, Instytut Psychologii, Uniwersytet Wrocławski (2014). p. 35. doi: 10.13140/2.1.4811.5521

[19] Gosling SD Rentfrow PJ Swann WB. A very brief measure of the Big-Five personality domains. J Res Pers. (2003) 37:504–28. doi: 10.1016/S0092-6566(03)00046-1

[20] John OP Srivastava S. The Big Five Trait taxonomy: History, measurement, and theoretical perspectives. In: Handbook of personality: Theory and research, 2nd ed. Guilford Press, New York, NY, US (1999). p. 102–38.

[21] Field A. Discovering statistics using IBM SPSS statistics. 6th. London: Sage publications limited (2024).

[22] Tabachnick BG Fidell LS. Using Multivariate Statistics. 7th. New York: Pearson (2019).

[23] Midway S Robertson M Flinn S Kaller M. Comparing multiple comparisons: practical guidance for choosing the best multiple comparisons test. PeerJ. (2020) 8:e10387. doi: 10.7717/peerj.10387 33335808 PMC7720730

[24] Olejnik S Algina J. Measures of effect size for comparative studies: applications, interpretations, and limitations. Contemp Educ Psychol. (2000) 25:241–86. doi: 10.1006/ceps.2000.1040 10873373

[25] Piłat-Borcuch M. Pomiędzy tożsamością osobową a postawą społeczną(2013). Available online at: http://yadda.icm.edu.pl/baztech/element/bwmeta1.element.baztech-30fb4e6a-ea41-4381-a351-b024b4d37499 (Accessed April 16, 2024).

[26] Satchidanand N Gunukula SK Lam WY McGuigan D New I Symons AB. Attitudes of healthcare students and professionals toward patients with physical disability: a systematic review. Am J Phys Med Rehabil. (2012) 91:533–45. doi: 10.1097/PHM.0b013e3182555ea4 22596075

[27] Stachura K Garven F. A national survey of occupational therapy students’ and physiotherapy students’ attitudes to disabled people. Clin Rehabil. (2007) 21:442–9. doi: 10.1177/0269215507073495 17613565

[28] Chan CCH Lee TMC Yuen H-K Chan F. Attitudes towards people with disabilities between Chinese rehabilitation and business students: An implication for practice. Rehabil Psychol. (2002) 47:324–38. doi: 10.1037/0090-5550.47.3.324

[29] Cooper AE Rose J Mason O. Mental health professionals’ attitudes towards people who are deaf. J Community Appl Soc Psychol. (2003) 13:314–9. doi: 10.1002/casp.725 15929231

[30] Orm S Blikstad-Blumenthal C Fjermestad K. Attitudes toward people with intellectual disabilities in Norway. Int J Dev Disabil. (2023) 0:1–7. doi: 10.1080/20473869.2023.2230825 PMC11843632 39990081

[31] Hamad Alnahdi G. The interaction between knowledge and quality of contact to predict Saudi university students’ attitudes toward people with intellectual disability. Int J Dev Disabil. (2019) 67:202–8. doi: 10.1080/20473869.2019.1638582 PMC8223900 34221362

[32] Paris MJ. Attitudes of medical students and health-care professionals toward people with disabilities. Arch Phys Med Rehabil. (1993) 74:818–25. doi: 10.1016/0003-9993(93)90007-w 8347067

[33] Tervo RC Azuma S Palmer G Redinius P. Medical students’ attitudes toward persons with disability: a comparative study. Arch Phys Med Rehabil. (2002) 83:1537–42. doi: 10.1053/apmr.2002.34620 12422321

[34] Duckworth SC. The effect of medical education on the attitudes of medical students towards disabled people. Med Educ. (1988) 22:501–5. doi: 10.1111/j.1365-2923.1988.tb00793.x 2976117

[35] Sahin H Akyol AD. Evaluation of nursing and medical students’ attitudes towards people with disabilities. J Clin Nurs. (2010) 19:2271–9. doi: 10.1111/j.1365-2702.2009.03088.x 20522157

[36] Chadd EH Pangilinan PH. Disability attitudes in health care: a new scale instrument. Am J Phys Med Rehabil. (2011) 90:47–54. doi: 10.1097/PHM.0b013e3182017269 21169745

[37] O’Donnell D. Use of the SADP for measurement of attitudes of Chinese dental students and dental surgery assistants toward disabled persons. Spec Care Dent. (1993) 13:81–5. doi: 10.1111/j.1754-4505.1993.tb01460.x 8272989

[38] Matziou V Galanis P Tsoumakas C Gymnopoulou E Perdikaris P Brokalaki H. Attitudes of nurse professionals and nursing students towards children with disabilities. Do nurses really overcome children’s physical and mental handicaps? Int Nurs Rev. (2009) 56:456–60. doi: 10.1111/j.1466-7657.2009.00735.x 19930074

[39] Wöhrle J Franke S Kissgen R. The German Multidimensional Attitude Scale Toward Persons With Disabilities (G-MAS): A factor analytical study among high-school students. Rehabil Psychol. (2018) 63:83–91. doi: 10.1037/rep0000170 28981307

[40] Lund E Seekins T. Early exposure to people with physical and sensory disabilities and later attitudes toward social interactions and inclusion. Phys Disabil Educ Relat Serv. (2014) 33:1. doi: 10.14434/pders.v33i1.4825

[41] Tsujita M Ban M Kumagaya S-I. The Japanese multidimensional attitudes scale toward persons with autism spectrum disorders1. Jpn Psychol Res. (2021) 63:129–39. doi: 10.1111/jpr.12298

[42] Domagała-Zyśk E. Attitudes of different age groups toward people with intellectual disability during the COVID-19 pandemic. Front Psychiatry. (2021) 12:591707. doi: 10.3389/fpsyt.2021.591707 34322039 PMC8310948

[43] Szumski G Smogorzewska J Grygiel P. Attitudes of students toward people with disabilities, moral identity and inclusive education—A two-level analysis. Res Dev Disabil. (2020) 102:103685. doi: 10.1016/j.ridd.2020.103685 32422515

[44] Benham PK. Attitudes of occupational therapy personnel toward persons with disabilities. Am J Occup Ther. (1988) 42:305–11. doi: 10.5014/ajot.42.5.305 2969193

[45] Arabi H Adarmouch L Ahmed Eladip G. The assessment of student doctors’ attitude towards disabled people after teaching them a module. Acta Bio Med Atenei Parm. (2021) 92:e2021059. doi: 10.23750/abm.v92i2.9547 PMC8182594 33988170

[46] Ryan TA Scior K. Medical students’ attitudes towards people with intellectual disabilities: A literature review. Res Dev Disabil. (2014) 35:2316–28. doi: 10.1016/j.ridd.2014.05.019 24952372

[47] Pettigrew TF Tropp LR. How does intergroup contact reduce prejudice? Meta-analytic tests of three mediators. Eur J Soc Psychol. (2008) 38:922–34. doi: 10.1002/ejsp.504

[48] Gething L. Attitudes toward people with disabilities of physiotherapists and members of the general population. Aust J Physiother. (1993) 39:291–6. doi: 10.1016/S0004-9514(14)60489-X 25026424

[49] Eberhardt K Mayberry W. Factors influencing entry-level occupational therapists’ attitudes toward persons with disabilities. Am J Occup Ther. (1995) 49:629–36. doi: 10.5014/ajot.49.7.629 7573333

[50] McManus JL Feyes KJ Saucier DA. Contact and knowledge as predictors of attitudes toward individuals with intellectual disabilities. J Soc Pers Relatsh. (2011) 28:579–90. doi: 10.1177/0265407510385494

[51] Keith JM Bennetto L Rogge RD. The relationship between contact and attitudes: Reducing prejudice toward individuals with intellectual and developmental disabilities. Res Dev Disabil. (2015) 47:14–26. doi: 10.1016/j.ridd.2015.07.032 26342326

[52] Cecchetti M Last J Lynch J Linehan C. Evaluating the longitudinal impact of a disability education intervention on medical students’ attitudes towards persons with a disability. Disabil Health J. (2021) 14:101092. doi: 10.1016/j.dhjo.2021.101092 33722577

[53] Albuquerque C P Pinto I G Ferrari L. Attitudes of parents of typically developing children towards school inclusion: the role of personality variables and positive descriptions. Eur J Spec Needs Educ. (2019) 34:369–82. doi: 10.1080/08856257.2018.1520496

[54] Krischler M Pit-ten Cate IM. Inclusive education in Luxembourg: implicit and explicit attitudes toward inclusion and students with special educational needs. Int J Incl Educ. (2020) 24:597–615. doi: 10.1080/13603116.2018.1474954

[55] Lisle K. Identifying the negative stigma associated with having a learning disability. Honors Theses. Bucknell University (US). (2011). 22. Available online at: https://digitalcommons.bucknell.edu/honors_theses/22 (Accessed December 16, 2024).

[56] Akbulut Zencirci S Metintas S Kosger F Melekoglu M. Impact of a mixed method training programme on attitudes of future doctors toward intellectual disability. Int J Dev Disabil. (2024) 70:261–7. doi: 10.1080/20473869.2022.2085023 PMC10930146 38481456

[57] Vuković A Jovanović OS Vranješević J Popovac A Perić T Marković D. Using role play to develop positive attitudes toward people with disabilities among dental students: An exploratory pilot study. Spec Care Dent. (2023) 43:806–14. doi: 10.1111/scd.12816 36572954

[58] Lin I-H Wang C-Y Lin Y-N Chen H-C Lin L-F. Simulation-based holistic education in physiotherapy interns to increase empathy toward older adults and individuals with disabilities. BMC Geriatr. (2022) 22:795. doi: 10.1186/s12877-022-03500-x 36224529 PMC9555068

[59] Albaum C Mills A Morin D Weiss JA. Attitudes toward people with intellectual disability associated with integrated sport participation. Adapt Phys Act Q APAQ. (2022) 39:86–108. doi: 10.1123/apaq.2021-0006 34728589

[60] Hayward L Fragala-Pinkham M Schneider J Coe M Vargas C Wassenar A. Examination of the short-term impact of a disability awareness training on attitudes toward people with disabilities: A community-based participatory evaluation approach. Physiother Theory Pract. (2021) 37:257–70. doi: 10.1080/09593985.2019.1630879 31204874

[61] Maor R. Profiles of peer-rejected individuals: their attitudes toward the intellectual disability population and the mediating role of resilience. J Genet Psychol. (2024) 185:323–36. doi: 10.1080/00221325.2024.2301943 38192068

[62] Perinelli E Gremigni P. Use of social desirability scales in clinical psychology: A systematic review. J Clin Psychol. (2016) 72:534–51. doi: 10.1002/jclp.22284 26970350

