# Supplementary material for: Attitudes towards persons with disabilities vs. personality traits of Polish students
Source: Front Psychiatry. 2025 Jan 27;15:1477877. doi: 10.3389/fpsyt.2024.1477877 (PMC11808036; doi:10.3389/fpsyt.2024.1477877)
Supplement: Supplementary file 1 [file Table1.docx]

# Supplementary material

**Table 1**

*Results from four general linear model analyses to predict the Multidimensional Attitudes Towards Persons with Disabilities (MAS) scores from scores on the Ten Item Personality Inventory (TIPI) scales and interaction with gender*

| MAS scores | *F* | *df* | *p* | *R_s_^2^* | Predictors | β | CI 95% | | *p* |
| --- | --- | --- | --- | --- | --- | --- | --- | --- | --- |
|  |  |  |  |  |  |  | *LL* | *UL* |  |
| Emotions | 13.80 | 3, 591 | <0.001 | 0.06 | Extraversion | -0.21 | -0.36 | -0.05 | 0.010 |
|  |  |  |  |  | Gender | 0.00 | -0.17 | 0.18 | 0.476 |
|  |  |  |  |  | Gender * Extraversion | -0.07 | -0.25 | 0.11 | 0.464 |
| Beliefs | 9.90 | 3, 591 | <0.001 | 0.04 | Extraversion | -0.16 | -0.32 | 0.00 | 0.041 |
|  |  |  |  |  | Gender | -0.18 | -0.36 | -0.01 | 0.970 |
|  |  |  |  |  | Gender * Extraversion | -0.06 | -0.24 | 0.13 | 0.551 |
| Behaviors | 11.70 | 3, 591 | <0.001 | 0.05 | Extraversion | -0.31 | -0.46 | -0.15 | <0.001 |
|  |  |  |  |  | Gender | -0.29 | -0.46 | -0.12 | 0.015 |
|  |  |  |  |  | Gender * Extraversion | 0.15 | -0.03 | 0.33 | 0.096 |
| Total | 20.00 | 3, 591 | <0.001 | 0.09 | Extraversion | -0.29 | -0.44 | -0.14 | <0.001 |
|  |  |  |  |  | Gender | -0.17 | -0.34 | 0.00 | 0.703 |
|  |  |  |  |  | Gender * Extraversion | -0.01 | -0.19 | 0.17 | 0.900 |
| Emotions | 11.30 | 3, 591 | <0.001 | 0.05 | Agreeableness | -0.24 | -0.39 | -0.09 | 0.002 |
|  |  |  |  |  | Gender | 0.04 | -0.13 | 0.21 | 0.993 |
|  |  |  |  |  | Gender * Agreeableness | 0.01 | -0.17 | 0.19 | 0.916 |
| Beliefs | 13.80 | 3, 591 | <0.001 | 0.06 | Agreeableness | -0.17 | -0.32 | -0.02 | 0.023 |
|  |  |  |  |  | Gender | -0.15 | -0.32 | 0.02 | 0.508 |
|  |  |  |  |  | Gender * Agreeableness | -0.09 | -0.27 | 0.08 | 0.298 |
| Behaviors | 20.00 | 3, 591 | <0.001 | 0.09 | Agreeableness | -0.35 | -0.49 | -0.20 | <0.001 |
|  |  |  |  |  | Gender | -0.24 | -0.41 | -0.07 | 0.091 |
|  |  |  |  |  | Gender * Agreeableness | -0.10 | -0.07 | 0.27 | 0.264 |
| Total | 24.60 | 3, 591 | <0.001 | 0.11 | Agreeableness | -0.33 | -0.47 | -0.18 | <0.001 |
|  |  |  |  |  | Gender | -0.12 | -0.29 | 0.05 | 0.770 |
|  |  |  |  |  | Gender * Agreeableness | 0.00 | -0.17 | 0.17 | 0.999 |
| Emotions | 5.21 | 3, 591 | 0.001 | 0.02 | Conscientiousness | -0.18 | -0.35 | -0.02 | 0.026 |
|  |  |  |  |  | Gender | 0.04 | -0.14 | 0.22 | 0.818 |
|  |  |  |  |  | Gender * Conscientiousness | 0.03 | -0.15 | 0.22 | 0.735 |
| Beliefs | 6.24 | 3, 591 | <0.001 | 0.03 | Conscientiousness | -0.17 | -0.32 | 0.00 | 0.052 |
|  |  |  |  |  | Gender | -0.15 | -0.33 | 0.03 | 0.681 |
|  |  |  |  |  | Gender * Conscientiousness | 0.00 | -0.18 | 0.19 | 0.979 |
| Behaviors | 12.20 | 3, 591 | <0.001 | 0.05 | Conscientiousness | -0.35 | -0.51 | -0.19 | <0.001 |
|  |  |  |  |  | Gender | -0.24 | -0.41 | -0.06 | 0.003 |
|  |  |  |  |  | Gender * Conscientiousness | 0.22 | 0.04 | 0.40 | 0.018 |
| Total | 11.30 | 3, 591 | <0.001 | 0.05 | Conscientiousness | -0.29 | -0.45 | -0.13 | <0.001 |
|  |  |  |  |  | Gender | -0.12 | -0.29 | 0.06 | 0.195 |
|  |  |  |  |  | Gender * Conscientiousness | 0.09 | -0.09 | 0.28 | 0.312 |
| Emotions | 2.33 | 3, 591 | 0.074 | 0.01 | Emotional stability | -0.10 | -0.27 | 0.06 | 0.211 |
|  |  |  |  |  | Gender | -0.03 | -0.22 | 0.15 | 0.983 |
|  |  |  |  |  | Gender * Emotional stability | -0.01 | -0.20 | 0.19 | 0.923 |
| Beliefs | 1.63 | 3, 591 | 0.184 | 0.00 | Emotional stability | -0.02 | -0.19 | 0.14 | 0.782 |
|  |  |  |  |  | Gender | -0.18 | -0.37 | 0.00 | 0.798 |
|  |  |  |  |  | Gender * Emotional stability | -0.03 | -0.22 | 0.15 | 0.719 |
| Behaviors | 4.88 | 3, 591 | 0.002 | 0.02 | Emotional stability | -0.17 | -0.33 | -0.01 | 0.039 |
|  |  |  |  |  | Gender | -0.32 | -0.51 | -0.13 | 0.002 |
|  |  |  |  |  | Gender * Emotional stability | 0.23 | 0.04 | 0.42 | 0.016 |
| Total | 2.30 | 3, 591 | 0.077 | 0.01 | Emotional stability | -0.13 | -0.29 | 0.04 | 0.127 |
|  |  |  |  |  | Gender | -0.20 | -0.39 | -0.01 | 0.250 |
|  |  |  |  |  | Gender * Emotional stability | 0.06 | -0.13 | 0.25 | 0.556 |
| Emotions | 13.70 | 3, 591 | <0.001 | 0.06 | Openness to experience | -0.33 | -0.48 | -0.18 | <0.001 |
|  |  |  |  |  | Gender | 0.07 | -0.10 | 0.24 | 0.331 |
|  |  |  |  |  | Gender * Openness to experience | 0.10 | -0.07 | 0.28 | 0.250 |
| Beliefs | 8.39 | 3, 591 | <0.001 | 0.04 | Openness to experience | -0.23 | -0.39 | -0.08 | 0.003 |
|  |  |  |  |  | Gender | -0.13 | -0.31 | 0.04 | 0.304 |
|  |  |  |  |  | Gender * Openness to experience | 0.07 | -0.11 | 0.25 | 0.471 |
| Behaviors | 12.20 | 3, 591 | <0.001 | 0.05 | Openness to experience | -0.23 | -0.38 | -0.07 | 0.004 |
|  |  |  |  |  | Gender | -0.23 | -0.41 | -0.06 | 0.426 |
|  |  |  |  |  | Gender * Openness to experience | 0.02 | -0.16 | 0.20 | 0.805 |
| Total | 19.90 | 3, 591 | <0.001 | 0.09 | Openness to experience | -0.36 | -0.51 | -0.21 | <0.001 |
|  |  |  |  |  | Gender | -0.09 | -0.26 | 0.08 | 0.202 |
|  |  |  |  |  | Gender * Openness to experience | 0.10 | -0.08 | -0.27 | 0.286 |

*Note*. Analysis was conducted for a set of 595 observations.

MAS – Multidimensional Attitudes Scale Towards Persons With Disabilities, *F* – ANOVA model fit test, *R_s_^2^* – coefficient of determination,
*p* – significance, *β* – standardised coefficient, *CI* – confidence interverbal, *LL* – lower limit, *UP* – upper limit
